# Advantages of an Automated Method Compared With Manual Methods for the Quantification of Intraepidermal Nerve Fiber in Skin Biopsy

**DOI:** 10.1093/jnen/nlab045

**Published:** 2021-05-26

**Authors:** Marta Francisca Corrà, Mafalda Sousa, Inês Reis, Fabiana Tanganelli, Nuno Vila-Chã, Ana Paula Sousa, Rui Magalhães, Paula Sampaio, Ricardo Taipa, Luís Maia

**Affiliations:** 1 From the Instituto de Ciências Biomédicas Abel Salazar (ICBAS), Universidade do Porto; 2 Department of Neurology, Centro Hospitalar Universitário do Porto (CHUP); 3 Instituto de investigação e inovação em Saúde da Universidade do Porto (i3S), Porto, Portugal; 4 Department of Medicine IV, Geriatrics, University Hospital, LMU Munich, Munich, Germany

**Keywords:** Automated method, Intraepidermal nerve fiber density, Skin biopsy

## Abstract

Intraepidermal nerve fiber density (IENFD) measurements in skin biopsy are performed manually by 1–3 operators. To improve diagnostic accuracy and applicability in clinical practice, we developed an automated method for fast IENFD determination with low operator-dependency. Sixty skin biopsy specimens were stained with the axonal marker PGP9.5 and imaged using a widefield fluorescence microscope. IENFD was first determined manually by 3 independent observers. Subsequently, images were processed in their Z-max projection and the intradermal line was delineated automatically. IENFD was calculated automatically (fluorescent images automated counting [FIAC]) and compared with manual counting on the same fluorescence images (fluorescent images manual counting [FIMC]), and with classical manual counting (CMC) data. A FIMC showed lower variability among observers compared with CMC (interclass correlation [ICC] = 0.996 vs 0.950). FIMC and FIAC showed high reliability (ICC = 0.999). A moderate-to-high (ICC = 0.705) was observed between CMC and FIAC counting. The algorithm process took on average 15 seconds to perform FIAC counting, compared with 10 minutes for FIMC counting. This automated method rapidly and reliably detects small nerve fibers in skin biopsies with clear advantages over the classical manual technique.

## INTRODUCTION

A punch biopsy of the skin is a safe and minimally invasive diagnostic procedure to access small-diameter nerve fibers in the human skin, and it is recommended for the assessment of small fiber neuropathy (SFN), which affects 0.1% of the general population (1–3). Intraepidermal nerve fiber density (IENFD) is determined by measuring the number of small nerve fibers crossing the dermal-epidermal junction (or intradermal line) and calculated per millimeter. Small nerve fibers are stained with a pan-axonal antibody (PGP9.5), a marker of both myelinated and unmyelinated axons of peripheral nerves, revealing both cutaneous nerve terminals and axonal degeneration (4). This analysis has the advantage of providing a continuous quantification of nerve loss, guaranteeing the evaluation of disease progression and treatment efficacy (5).

Given its high sensitivity and specificity, in the past decade IENFD has become a widely recognized technique in clinical practice and is increasingly recommended to complement physical and neurophysiological evaluation in the study of SFN patients (6). In order to standardize the use of skin biopsy in clinical practice, the European Federation of Neurological Societies (EFNS) and the Peripheral Nerve Society created a task force to define the main guidelines for this methodology: tissue processing (biopsy collection, sample preparation, and sectioning) was listed, but staining procedures and IENFD quantification methods were not standardized, generating variability (7).

The quantitative determination of IENFD is performed manually by 1–3 operators (3 observers are recommended). Considering that the quantification is operator-dependent, it can result in a high interrater variability. Possible reasons for high variances among observers include: (i) the difficulty of identifying the intradermal line, which often looks blurred and not bright; (ii) the nerve fiber visualization on the maximum projection, which can generate confusion during counting; and (iii) the loss of fluorescence signal within months can affect the analysis results if repeated over time.

Previous reports investigated the interrater variability of IENFD quantification. Studies focusing on variability between 2 observers found an intraclass correlation coefficient (ICC) of 0.86–0.98 (8–10), indicating a high-reliability level. Other studies have shown significant differences in IENFD counting among 3 observers and questioned the reliability of the manual counting using a more accurate statistical analysis (11).

The classic technique is thus time- and human resources-consuming, limiting its use in the clinical setting. Therefore, it is necessary to investigate more standardized and less operator-dependent approaches for IENFD counting to improve reliability and standardize procedures both in research and clinical routine. With this in mind, some computerized strategies have been proposed (12), but they are generally not completely automated (4, 13), or involve private and high-cost software (14).

The lack of efficient and standardized tools for IENFD counting led us to develop a custom-made approach to achieve a feasible and reliable small fiber quantification method. For this purpose, we developed a novel two-step procedure for an automated and standardized IENFD quantification in skin biopsies: (i) a new approach for image digitization that allows a systematic identification of the intradermal line and therefore reduces variability among observers; and (ii) an algorithm for automated nerve fiber counting and IENFD measurement on fluorescence images.

This approach provides a freely available and less operator-dependent procedure to be applied in research and clinical practice. The resulting work will solve the lack of manual diagnostic accuracy and apply the new method in clinical practice.

## MATERIALS AND METHODS

### Patients

Skin biopsies from a pool of randomly selected subjects from Centro Hospitalar Universitário do Porto (CHUP) were included in the study. All participants gave their written informed consent for the study, which was approved by the local Ethics Committee of the University Hospital of Porto (PT) in accordance with the Helsinki Declaration.

### Skin Biopsy and Staining

Skin specimens were taken with a disposable 5-mm circular punch under sterile technique after topical anesthesia with lidocaine and no suture was needed. The anatomical sites of skin biopsies were the lateral side of the distal leg (10 cm above the malleolus) and the proximal thigh (20 cm below the greater trochanter).

After fixation in 4% paraformaldehyde, specimens were incubated in 10% Saccharose at 4°C overnight, then frozen with 2-Methylbutan. Immunohistochemical labeling was performed on 50-μm frozen sections using rabbit polyclonal protein-gene-product (PGP9.5) antibody (Zytomed Systems, Berlin, Germany; 1:250). Indirect immunofluorescent technique with Cyanine 3 (Jackson ImmunoResearch Laboratories, West Grove, PA; 1:50) as fluorescent secondary antibody was performed. The nuclei were stained with Vectashield antifade mounting medium with DAPI. Stained sections were stored at –20°.

### Biopsy Fluorescence Image Acquisition

The same skin specimens were then imaged using a motorized widefield fluorescence microscope equipped with an HC PL FLUOTAR L 40x/0.60 objective (Leica DMI6000, Leica Microsystems). The nuclei were stained with DAPI (AT—Excitation: 340–380; BS: 400; Emission: 425 LP), and the rabbit polyclonal protein-gene-product (PGP9.5) antibody coupled with the Cy3 was used as a pan-axonal marker (TX2 Excitation: 540–580; BS: 595; Emission: 607–683). A Z-stack was acquired with a step size of 695 nm. The stack’s upper and lower limits were defined to include all fibers from the epidermis and dermis. Images were acquired with a Hamamatsu Flash 4.2 sCMOS camera in mode binning 2 × 2, and the stitching was done within the LAS X Navigator extension.

### Intraepidermal Nerve Fiber Density

IENFD determinations were performed and compared under 3 different conditions: (i) a classical manual counting (CMC) technique on live skin biopsy sections by 3 observers; (ii) manual counting by the same 3 observers of the fluorescence images (FIMC) acquired with a fluorescence microscope and manually counted with Fiji drawing tools; and (iii) automated counting of the same fluorescence images (FIAC) by a developed algorithm. The counting methods are described in detail below.

#### Classical Manual Counting

IENFD was determined manually for each specimen by counting directly through the oculars and focusing through the optical planes by 3 independent observers trained following published counting guidelines. Only single IENFD crossing the intradermal (dermal-epidermal) junction was counted (7). All the tissue sections were analyzed using Nikon Eclipse E400 fluorescence microscope at 40× high magnification. No image acquisition was performed. The length of the section was measured using the 2.5× objective and LAS V4.3 software. Fibers density was calculated as the number of IENFD per length of the section (IENFD/mm).

#### Fluorescence Images Manual Counting

A manual intradermal line (MIL) was drawn by one of the observers using Fiji drawing tools by following the epidermal cells stained in the DAPI channel. Fibers intersecting the MIL were manually counted by the same observers of the classical technique. A counting quality control was made by annotating the counted fibers with the Fiji point tool and the images were saved for further validation (15).

#### Fluorescence Images Automated Counting

**FIGURE 1.**
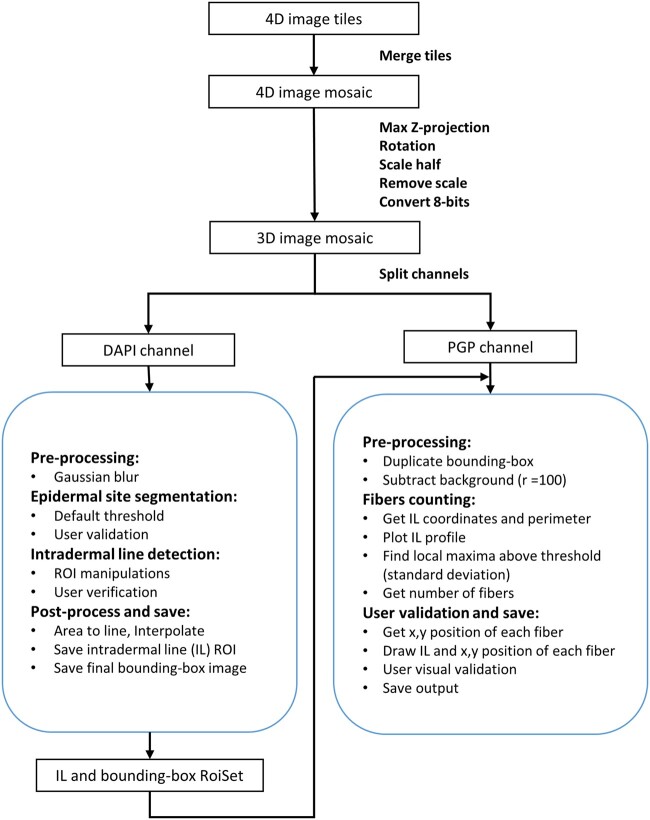
Automatic IENFD counting workflow.

Each stack of images was used to measure the PGP9.5 fluorescent fibers within the whole skin section and its Z maximum (Z-max) projection. Two open-source Fiji macro scripts were written to detect the intradermal line and perform automated IENFD quantification. Although the analysis was fully automatic, the scripts were written to have full user interaction, control, and validation of the main steps. The algorithm is schematized in Figure 1 and consisted of the following steps:

### Input Images

After microscope acquisition, images are prepared and preprocessed for further analysis. (a) Each image stack is merged to obtain the full skin section into a 4D mosaic image (XYZC). (b) A scaling factor of 0.5 is applied to all images since the original image has a nonworkable size (∼20 Gb each). (c) A maximum Z-projection of focus planes is applied to reduce z stacks into a 3D mosaic image (XYC). (d) 8-Bit conversion and scale removal are done to apply always the same range of values in the following steps. (e) Images should be oriented with the epidermal site in a right-down direction, so the user is prompted to correct image rotation if necessary. (f) Images are split into 2 different channels: the DAPI channel is used for the Intradermal line detection and the PGP channel to count the IENFD.

### Automated Intradermal Line Detection

DAPI channel automatically delineates the intradermal line following the cell nuclei reference stained in the epidermal site. (a) Correct DAPI channel illumination and apply a Gaussian blur filter of sigma 20 px to enhance the epidermal site. (b) In some cases, the full section was cropped in continuous regions of interest (ROIs) to overcome intensity variabilities or mounting issues. Each ROI was analyzed independently and the result was summed up at the end. (c) The epidermal site segmentation is done by applying a default threshold. The user is prompted to validate and, if necessary, manually adjust for better results. The final ROI is segmented if the area is larger than 5000 px, with no holes and without touching the image’s edges. (d) Some ROI manipulations are done to select the intradermal line automatically (cut the ROI extremities with a shrunk bounding-box [Enlarge –10 px]; select the upper line; area to line; interpolate). The user is prompted to validate the final intradermal line. (e) The intradermal line (AIL) and the bounding-box are saved as a roiSet with the same name as the image file.

### Automated IENFD Counting

The number of fibers crossing the intradermal line is quantified as the local maximal peaks of the PGP channel’s fluorescence signal. (a) Remove the PGP channel’s background noise with the subtract background function (rolling ball radius of 100 px). Apply the bounding-box saved in the previous step to get the correct intradermal line position. (b) Get the (*x*, *y*) coordinates of the AIL and measure the perimeter by multiplying with pixel size to get the correct intradermal line length (in millimeters). (c) Plot AIL profile and find the local maxima above the threshold value that is, by default, the profile’s standard deviation. The user is prompted to change the threshold value if necessary. (d) The (*x*, *y*) coordinates of each fiber crossing the AIL are counted and drawn in the image for further validation. (e) A validation step with user visualization of the result can be done, allowing the repetition of steps (c) and (d) until satisfied. (f) Image with AIL drawn and fiber’s crossing and the Log file with numeric results are saved.

### Validation of IENFD

IENFD is calculated as the number of IENFD per length of the section (IENFD/mm). IENFD was validated by 3 methods: (i) validation of IENFD FIMC with the CMC; (ii) validation of FIAC with the FIMC; (iii) validation of the AIL detection with the MIL drawing. In the first method, the sections length used for normalization was obtained in the classical technique. In the second method, the section’s length was obtained from the intradermal line perimeter drawn manually. The automatic intradermal line length was compared with the manual drawn intradermal line length in the last method.

### Statistical Analysis

The ICC (2-way mixed average measures [consistency]) and the relative intertrial variability were calculated to determine the interrater variability among the 3 observers during CMC and FIMC. Relative intertrial variability was expressed as the percentage obtained from dividing the difference between the 2 values by the mean value. Relative intertrial variability values of <10% indicate a high degree of reproducibility (10). The accuracy between each pair of observers was estimated by performing a correlation analysis (Pearson or Spearman, based on sample distributions) and the coefficients of variation (or relative standard deviation).

To compare the manual counting method with the automated counting algorithm, ICC, correlation analysis (*r*), paired comparison and coefficients of variation were calculated. Bland-Altman plots were used to evaluate the agreement between the 2 techniques (16).

All statistical analysis was performed using the SPSS 25 software package, and results were expressed in mean ± SD. p values < 0.05 were considered statistically significant.

## RESULTS

A total of 60 skin biopsy specimens from a total of 10 participants with no known diagnosis of SFN were analyzed. The mean age of the subjects was 69.9 years old (20% F). IENFD on live sections with CMC technique was 8.3 (3.4).

### Image Processing and Automated IENFD

**FIGURE 2.**
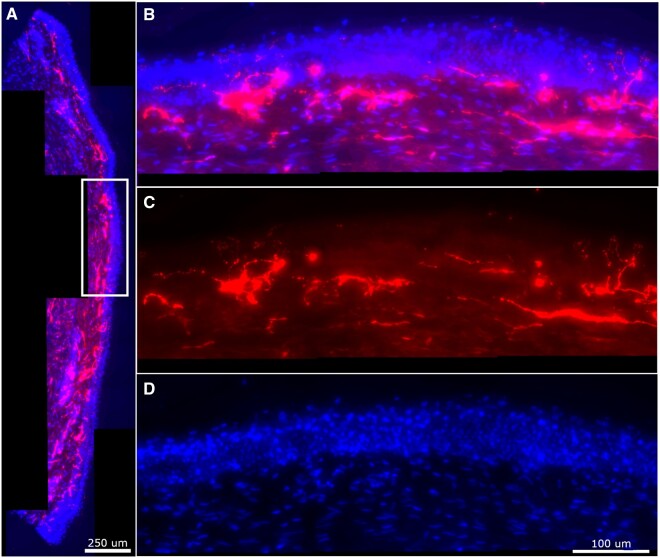
Original biopsy section obtained in a widefield fluorescence microscope, 40×/0.60 objective. **(A)** A middle z-plane of the 4-dimensional section. **(B, C)** Rotation of the white square in panel **A**. **(B)** Maximum Z-projection; **(C)** maximum Z-projection of the PGP channel; **(D)** maximum Z-projection of the DAPI channel.

The following section describes the automated IENFD and intradermal line detection result in the fluorescence skin biopsy images. Figure 2 illustrates a full biopsy section with PGP fluorescence highlighting the nerve fibers and DAPI fluorescence staining the epidermal site cell nucleus. Tissue thickness allowed the acquisition of images with around 30 z-focused-planes. With maximum z-projection of the middle 30 z-planes, each fiber's signal is kept and, in most of the cases, enhanced. Figure 1C, D illustrates each channel information separately.

**FIGURE 3.**
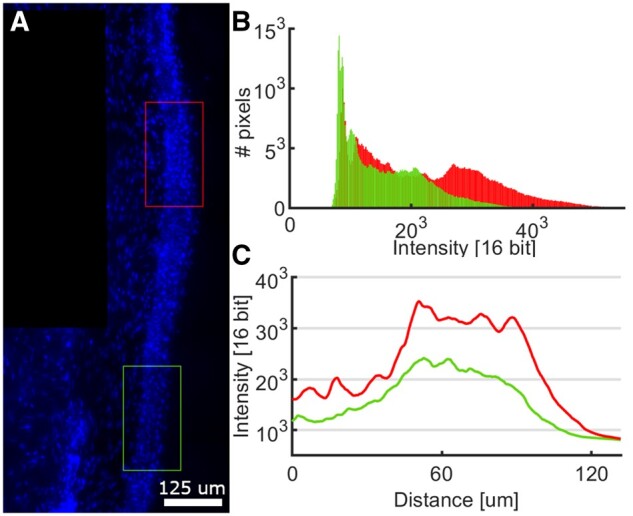
Intravariability of the staining fluorescence. **(A)** A cropped section from the DAPI channel of a biopsy section; the red square delimitates a region with high-intensity values compared with the green region with low-intensity values. **(B)** Histograms of intensity value distribution from each region. **(C)** Plot profiles from each region.

A high portion of the acquired images presented a good signal-to-noise ratio. Images also presented a high variability of the intensity values, not only between samples but also within the same sample, as illustrated in Figure 3. Different intensity histograms compromise the automatic segmentation process necessary in the detection of the epidermal site. Therefore, some images were cropped in continuous regions and analyzed separately to have less intensity variability in each region. Mounting the tissue on the coverslip could generate samples in which the biopsies' tips were slightly raised concerning the rest of the tissue. In those cases, the images were cropped to obtain most of the tissue of interest and remove the tissue tips from the analysis.

**FIGURE 4.**
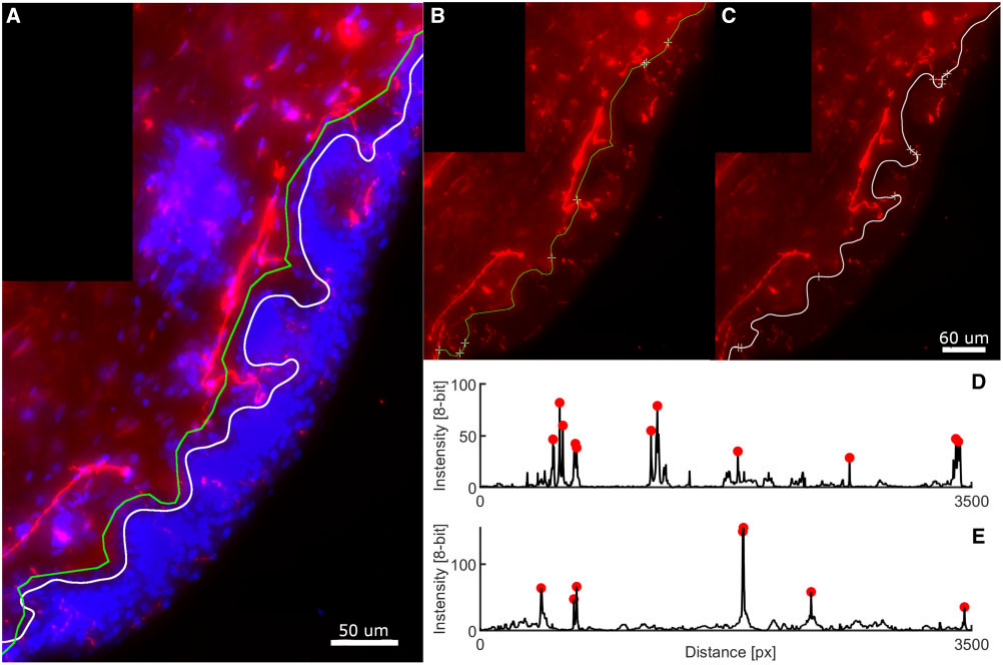
Automatic intradermal line detection and IENFD quantification. **(A)** Cropped section from a biopsy image of the automatic (AIL) and manual (MIL) intradermal lines (AIL in white and MIL in green). **(B, C)** Panels illustrate the detected IENFD spots crossing AIL and MIL, respectively. **(D, E)** Intradermal line profiles of AIL and MIL, respectively, with crossing fibers detected as the local maximum values (threshold = 20 [in an 8-bits range], minimum distance = 10 px).

The automatic intradermal line detection was achieved in all the images, and each length was qualitatively compared with the MIL. Figure 4 illustrates the comparison of both manual and automatic intradermal lines. MIL drawing was more rectilinear, while the AIL detection followed the epidermal site scrupulously on top of the epidermal site. As a result, the total AIL length was significantly bigger than MIL length (p < 0.001) (Supplementary Data  Fig. S1), and the number of fibers intersecting both intradermal lines was consequently different. These differences were therefore normalized applying a correction factor of 1.2 (±0.2), which allowed the use of both MIL and AIL in the IENFD counting. Nevertheless, in order to compare the automated and the manual counting as accurately as possible, MIL was used as a fixed variable for both counting methods in the following comparisons. The total algorithm process took on average 15 seconds to perform the FIAC counting (depending on the image size, but not on the number of fibers).

### Interrater Variability Between Observers During Manual Counting

We compared the interrater variability between observers either in the CMC or FIMC.

#### Classical Manual Counting

The main descriptive characteristics of the 3 observers’ counting are described in Supplementary Data  Table S1. Interclass correlation (ICC) among the observers was 0.950 (Table 1). The coefficient of variation among the observers was 14.7%. In terms of operator time, manual counting took on average 10 minutes per section for each operator.

**TABLE 1.** Interrater Variability Among 3 Observers for Both Techniques

	CMC		FIMC	
	ICC = 0.950		ICC = 0.996	
	*r*	RIV (SD)	*R*	RIV (SD)
Observer 1 and 2	0.808***	23.4(16)%	0.966***	8.8(8)%
Observer 1 and 3	0.875***	19.4(14)%	0.949***	11.2(9)%
Observer 2 and 3	0.942***	13.7(12)%	0.948***	10(10)%

CMC, classical technique manual counting; FIMC, fluorescence images manual counting; ICC, interclass correlation; RIV, relative intertrial variability.

Degrees of correlation (*r*) and relative intertrial variability (RIV) of manual counting among 3 using the classical technique and fluorescence images.

*** p < 0.001.

#### Fluorescence Images Manual Counting

The same 60 skin biopsy specimens were acquired with a multispectral camera-based fluorescence Leica microscope, as described. After the image preprocessing and manually drawing the intradermal line, the IENFD was manually counted by the same 3 observers (Supplementary Data  Table S1). ICC among the observers was 0.996 (Table 1). The coefficient of variation among observers was 8.1%. The FIMC showed significantly lower variability among observers compared with the CMC method. FIMC was comparable with the CMC counting times: it took on average 10 minutes to draw the line and manually count the fibers.

### Comparison of the Methods

#### FIAC Versus FIMC Methods

**FIGURE 5.**
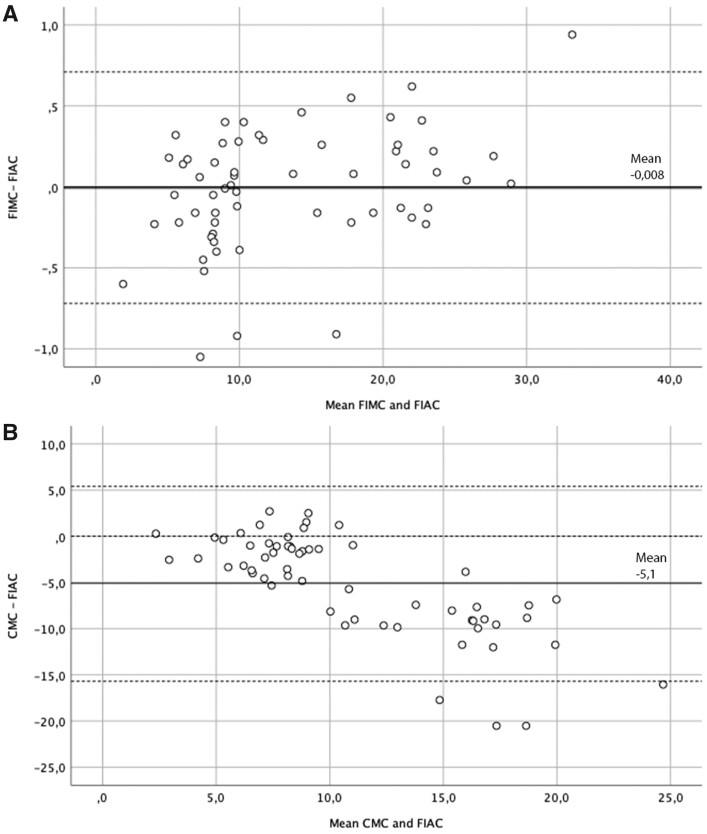
Agreement analysis between the counting methods. The *X*-axis represents the difference between the 2 methods and *Y*-axis is the mean of the 2 methods. **(A)** Agreement analysis between FIMC and FIAC. The bias of –0.008 units (bold line) is represented by the gap between the *X*-axis, corresponding to zero differences, and the parallel line to the *X*-axis at –0.008 units. **(B)** Agreement analysis between CMC and FIAC. The bias is –5.47 units. The limits of agreement are represented in dotted lines in both figures.

After demonstrating that manual counting on fluorescence images showed high reliability and decreased variability among observers, FIMC and FIAC methods were compared with validate the automated algorithm (Table 2). ICC between the 2 counting methods was 0.999. Correlation analysis showed a significant and robust correlation between the 2 methods (*r* = 0.995; p < 0.001). The mean coefficient of variation was 2.1%; no significant differences were shown between counting mean values (p = 0.817). Bland-Altman plots (Fig. 5A) represented a strong agreement between manual and automated counting, confirmed by a mean difference of –0.008 units between the 2 counting methods (16).

**TABLE 2.** IENFD Using 3 Distinctive Counting Methods

	CMC	FIMC	FIAC
Mean (SD)	8.3 (3.4)	13.5 (7.4)	13.5 (7.2)
95% confidence interval	(7.4–9.2)	(11.5–15.4)	(11.6–15.3)
Median	7.7	9.7	10
IQR	(5.9–10.8)	(8–20.9)	(8.2–20.6)

CMC, classical technique manual counting; FIMC, fluorescence images manual counting; FIAC, fluorescence images automated counting; IQR, interquartile range.

#### FIAC Versus CMC Methods

Results from the FIAC method were finally compared with the CMC method with the aim to apply the new method in clinical practice (Table 2). A moderate-to-high ICC between the 2 methods was observed (ICC = 0.705) and a significant degree of correlation (*r* = 0.651; p < 0.001). The coefficient of variation was 31.6% (Fig. 5B) and showed moderate agreement between the counting techniques, observed in a mean difference of –5.1 units.

**FIGURE 6.**
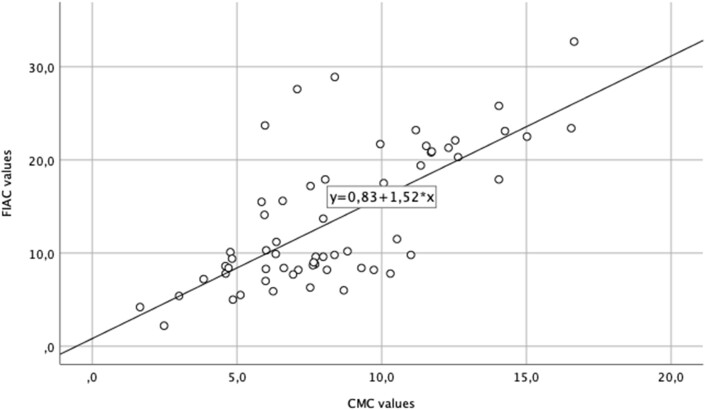
Regression analysis between manual and automated counting. The black line represents the best-fit regression. A normalization of 0.83 was found through the linear regression slope.

Lastly, single IENFD values from both CMC and FIAC counting methods were compared with evaluate the new counting method's application in clinical practice. To define a normalization formula, we compared the FIAC results with the mean observer CMC results. Normalization is applied such that the automated detection rate becomes an indicator of IENFD, applicable in clinical routine. An initial regression analysis considered all the 60 biopsies and the coefficient was adjusted based on its variability for all the skin biopsy counts. Figure 6 shows the final regression line (blue line) for all the biopsies with a slope of 0.83 and *y*-intercept of *b* = 1.52. A standard error of 0.2 confirmed the feasibility of the normalization for this sample size (Fig. 6).

## DISCUSSION

IENFD quantification in skin constitutes an excellent method to investigate SFN (7, 17, 18). This methodology consists of specialized manual procedures of staining and counting, which require laborious methodological skills and training, and therefore prone to human error when applied in conventional laboratories (19). Here, we describe for the first time an automated method for IENFD in skin biopsies using fluorescence images acquired in widefield microscopes. This method automatically obtains the nerve fiber density estimation quickly and reliably from PGP’s axon fluorescence in the epidermis and dermis instead of observer-dependent visualization (20). We believe that this method has a high potential for clinical application.

One of the major advantages of the method is the use of free and open-source software; the ImageJ/Fiji program, a widely used software for microscope fluorescent image analysis (21). Fiji is supported by many online tutorials, allowing low complexity, reliability, and reproducibility with potential applicability for further quantification analysis (13). Other laboratories can use the developed algorithms to standardize the IENFD methodology or it can be easily adapted and upgraded depending on the requirements. In addition, any type of brightfield microscope and related software can be used to perform the acquisition and quantification, which confirms this method's general applicability in research and clinical facilities.

The digital long-term storing of patient data and information has recently become available in hospitals and pathology laboratories. The proposed automated method saves and stores this information to patients’ folders and shares it with other operators, favoring second opinions, and reliability.

Moreover, this new approach provides an easy workflow for clinicians and researchers. It enables quantifying IENFD of a skin section in ∼15 seconds (depending on the image size, not on the number of fibers nor biopsy site), allowing a faster quantification compared with manual counting (∼10 minutes), and improving what was proposed by Seger et al (14), where the average time needed for IENFD of one section was ∼3 minutes.

Overall, the user-friendly characteristics (e.g. the easy workflow and the quick repeatability), the reduction in processing time, and the significant degree of correlation with manual counting results (for comparisons over time) are the main advantages of the proposed method. Images can also be easily stored without losing intensity over time compared with the operator-dependent manual classification technique.

### Methodological Considerations

There are several methodological considerations to highlight. First, we performed indirect immunofluorescence staining of small nerve fibers with PGP9.5 antibody instead of other techniques, such as immunohistochemistry visualization. The main reason for this choice is that immunofluorescence makes easier identification of the exact point where fibers cross the intradermal junction, allowing more accurate IENFD counting than immunohistochemistry. Additionally, this technique allows 3D analysis via fluorescence microscopy with higher resolution, making it suitable for detecting smaller variations (18). Fluorescent staining techniques are therefore the preferred choice for the development of new automated counting methods, allowing better accuracy and sensitivity. In addition, in the last decades immunofluorescence has been widely used in clinical routine. On the other hand, fluorescence vanishes over time, and for this reason previous studies focused on more stable and conventional immunoperoxidase staining for IENFD (4, 7). Although both techniques allow a useful IENFD, we opted for immunofluorescence since small nerve fiber staining after formalin fixation might be discontinuous, inducing less accurate results.

DAPI staining of cell nuclei was considered as a reference for delineating the intradermal line. This choice was innovative and driven because the intradermal junction is often blurred due to thick skin biopsy sections of 50 μm, resulting in out-of-focal-plane fluorescence signals. To find a proper way to detect the intradermal junction automatically, we opted to use an anatomical reference that was easy to stain and detect. This choice was corroborated by previous reports considering epidermal cell nuclei as the dermal-epidermal reference (19, 22). We proposed a validated (compared with MIL detection) and AIL in order to efficiently and objectively quantify IENFD quickly and reliably, improving the manual drawing approach proposed in previous works (13).

Second, 2 technical aspects regarding preprocessing images need to be highlighted. We applied a maximum Z-projection in order to collect information from all stacks. This choice was justified by the necessity to gather multiple images taken at different focal distances to ensure a greater field of depth. Previous studies adopted different techniques: Tamura et al (23) set a fixed number of 32 “layers-images” for each biopsy section, while Seger et al (14) acquired 21 z-planes separated by 2 µm. We believe that this methodology allows better detection of nerve fibers in the z-plane and increases image details as Casanova-Molla reported (13). These methodological aspects allowed us to reduce the variability between observers during FIMC (ICC = 0.996; Table 1), compared with CMC.

Another technical observation is the possibility to manually select the brightness threshold during FIAC, which is also carried out in similar studies (13, 24). The most common automatization problem is distinguishing artifactual features from actual nerve fibers (25). Therefore, for a meaningful quantification of the fluorescent structures, the operator can select an appropriate threshold to avoid false positives or artifacts and ensure accuracy through operator validation. Manual threshold adjustments provided consistent measures in our sample, corresponding to very high reliability and low variability, as observed in the strong ICC and correlation values between FIMC and FIAC (ICC = 0.999 and not a significant difference mean values), also visually confirmed by the Bland-Altman plot (Fig. 5A).

Concerning the clinical application, a fairly significant correlation was observed between the automated method and the manual counting with live visualization (*r* = 0.651; p < 0.001). This is in line with results from other studies (13, 22), which also showed a similar correlation when comparing their methodologies with manual counting. However, despite the significant correlation, our technique showed a significant difference in mean values with the classic counting, more pronounced in samples with less fibers (Fig. 5B). This can be justified by the different nature of the 2 counting methods: one technique is based on the live visualization of small nerve fibers on biopsy sections, while the automated method consists of acquiring and preprocessing images; the specific characteristics, such as z-projections and fixed intradermal line definition can increase the number of small nerve fiber detection.

In light of these differences, a correction factor of 0.8 was calculated to apply the new method in clinical practice. Even though the correction factor showed small variability (SD = 0.2), it can be considered a promising preliminary step to be proved and adjusted in further analyses and with increased sample size.

Some limitations need to be addressed. Fluorescence immunostaining with PGP9.5 and DAPI for cell nuclei may have a disadvantage with respect to image quality, which could lead to lack of reproducibility. Two main factors may contribute: first, immunofluorescence vanishes over time. We recommend performing staining and image acquisition in <6 months to ensure optimal conditions for the efficacy of the automated method. Second, we used a free-floating staining approach, which may lead to discontinuity, especially for large biopsy sections. This approach was preferred over mounting sections directly on glass slides because it allows a better antibody penetration and thus should be the method of choice when thicker sections are used (such as 50 µm thickness of our samples) (26). To solve quality discontinuity in some images, we analyzed them into parts and subsequently summed up the counting results. Furthermore, the costs of motorized microscopes with automated acquisition set-ups are higher than traditional fluorescence microscopes. Still, costs are variable and depend on the type of set-up chosen (type of camera, number of filters, number of objectives). However, increased costs are counterbalanced by several advantages, including better accuracy, sensitivity and reproducibility of images. Automated multispectral camera-based microscopes give brighter and more defined images and allow more easily developed new counting methods and new techniques that could also be useful in clinical routine. Overall, the higher initial investment can provide faster acquisition time, reduction of human error and the possibility for the operator to perform other different tasks at the same time. Future work will be needed to focus on the reproducibility of counting within and between different neuropathological institutions and on implementing the automated counting algorithm with deep learning techniques.

In conclusion, we have developed a method to rapidly and reliably detect small nerve fibers in skin biopsies that can be applied in biomedical research as well as clinical settings. We demonstrated that this technique first acquires well-defined fluorescence images and automatically detects the intradermal line, reducing variability among observers during manual counting. We also developed a new algorithm for automated detecting fibers, easy and quick to use, which showed strong reliability and feasibility compared with manual counting. Additionally, a preliminary normalization of values demonstrated possible applicability and comparability of the method with the classical manual technique, allowing its application in clinical settings and diagnosis. We suggest this method as a complementary approach to classical determination of IENFD raising the efficacy for a more complete and standardized diagnostic tool for SFN.

## Supplementary Material

nlab045_Supplementary_Data

## Notes

The study was supported by Keep Control from the EU’s Horizon 2020 (H2020) research and innovation program under the MSCA-ITN-ETN grant agreement number 721577.

The datasets used and analyzed during the current study are available from the corresponding author on reasonable request.
