# Supplementary material for: Advantages of an Automated Method Compared With Manual Methods for the Quantification of Intraepidermal Nerve Fiber in Skin Biopsy
Source: J Neuropathol Exp Neurol. 2021 May 26;80(7):685–94. doi: 10.1093/jnen/nlab045 (PMC8357338; doi:10.1093/jnen/nlab045)
Supplement: nlab045_Supplementary_Data [file nlab045_supplementary_data.pdf]

Supplementary Figure 1.

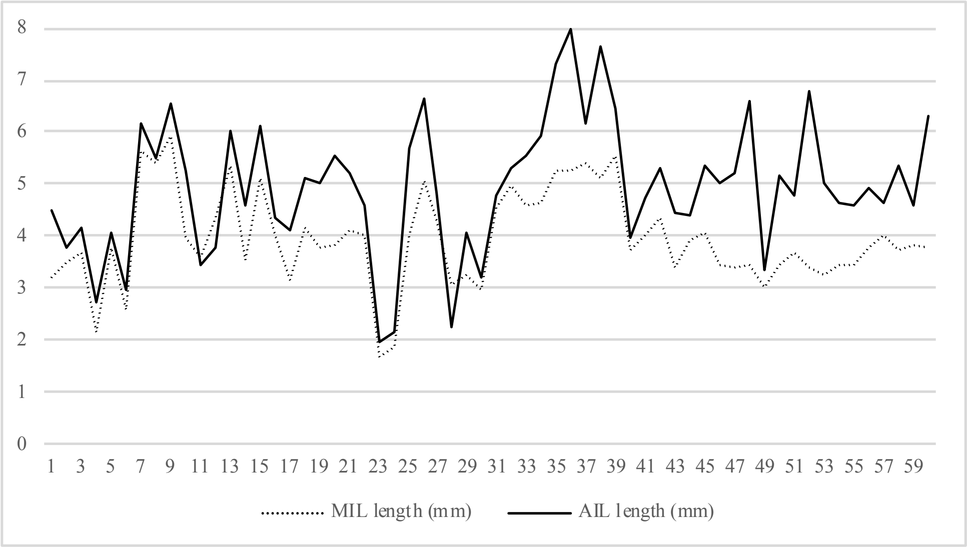

Supplementary Figure 1. Manual intradermal line (MIL) and automated intradermal line (AIL) length values for all skin biopsy cases (N=60). Lengths are expressed in mm. AIL lengths were significantly greater than MIL values (p<0.001).

Supplementary Table 1. Descriptive characteristics of IENFD manual counting among the three observers using the classic technique and the fluorescence images.

	**Observer 1**	**Observer 2**	**Observer 3**	**Total**
		**Classical technique (CTMT)**		
Mean (SD)	7.7 (2.9)	8.7 (3.8)	8.6 (3.8)	8.3 (3.4)
95% Confidence Interval	[6.9-8.4]	[7.7-9.7]	[7.6-9.6]	[7.4-9.2]
Median	6.8	7.8	8	7.7
IQR	[5.6-9.9]	[6-11]	[5.8-10.7]	[5.9-10.8]
		**Fluorescence images (FIMC)**		
Mean (SD)	13.7 (7.6)	13.7 (7.4)	13 (7.3)	13.5 (7.4)
95% Confidence Interval	[11.7-15.7]	[11.8-15.6]	[11.1-14.9]	[11.5-15.4]

Median	10.7	10.4	9.5	9.7
IQR	[8-20.8]	[7.6-20.6]	[7-18.9]	[8-20.9]

IQR: interquartile range.
